# The Surveillance of Chikungunya Virus in a Temperate Climate: Challenges and Possible Solutions from the Experience of Lazio Region, Italy

**DOI:** 10.3390/v10090501

**Published:** 2018-09-14

**Authors:** Francesco Vairo, Carlo Di Pietrantonj, Chiara Pasqualini, Alessia Mammone, Simone Lanini, Emanuele Nicastri, Concetta Castilletti, Federica Ferraro, Virginia Di Bari, Vincenzo Puro, Paola Scognamiglio, Antonino Di Caro, Maria Rosaria Capobianchi, Giuseppe Ippolito

**Affiliations:** 1 Regional Service for Surveillance and Control of Infectious Diseases (SERESMI)—Lazio Region, National Institute for Infectious Diseases “Lazzaro Spallanzani” IRCCS, 00149 Rome, Italy; francesco.vairo@inmi.it (F.V.); alessia.mammone@inmi.it (A.M.); federica.ferraro@inmi.it (F.F.); virginia.dibari@inmi.it (V.D.B.); vincenzo.puro@inmi.it (V.P.); paola.scognamiglio@inmi.it (P.S.); 2 Regional Service for Surveillance and Control of Infectious Diseases (SEREMI)—Regione Piemonte, 15121 Alessandria, Italy; cdipietrantonj@aslal.it (C.D.P.); cpasqualini@aslal.it (C.P.); 3 National Institute for Infectious Diseases “Lazzaro Spallanzani” IRCCS, 00149 Rome, Italy; emanuele.nicastri@inmi.it (E.N.); concetta.castilletti@inmi.it (C.C.); antonino.dicaro@inmi.it (A.D.C.); maria.capobianchi@inmi.it (M.R.C.); giuseppe.ippolito@inmi.it (G.I.)

**Keywords:** Chikungunya, surveillance, temperate climate, Italy

## Abstract

CHIKV has become an emerging public health concern in the temperate regions of the Northern Hemisphere as a consequenceof the expansion of the endemic areas of its vectors (mainly *Aedes aegypti* and *Aedes* *albopictus*). In 2017, a new outbreak of CHIKV was detected in Italy with three clusters of autochthonous transmission in the Lazio Region (central Italy), in the cities of Anzio, Rome, and Latina and a secondary cluster in the Calabria Region (south Italy). Given the climate characteristics of Italy, sporadic outbreaks mostly driven by imported cases followed by autochthonous transmission could occur during the summer season. This highlights the importance of a well-designed surveillance system, which should promptly identify autochthonous transmission. The use of a surveillance system integrating different surveillance tools, including entomological surveillance in a one health approach, together with education of the health care professionals should facilitate the detection, response, and control of arboviruses spreading.

## 1. Introduction

Chikungunya Virus (CHIKV) is a mosquito-borne alphavirus associated with a systemic self-limiting disease with a wide range of clinical presentations. CHIKV was firstly described during an outbreak in southern Tanzania in 1952–1953 [1]. The virus usually circulates in a sylvatic cycle. In urban epidemics, CHIKV can be transmitted to humans through the infectious bites by *Aedes* spp. mosquitoes [2]. Since 2000, the virus was found as one of the main causes of mosquito-borne infection in tropical and subtropical regions [3,4]. CHIKV has become an emerging public health concern in the temperate regions of the Northern Hemisphere as consequence of the expansion of the endemic areas of its vectors (mainly *Aedes aegypti* and *Aedes albopictus*) [5]. CHIKV has evolved in three major genotypes: West African, East/Central/South African (ECSA), and Asian [6,7]. A new variant of the ECSA genotype carrying a mutation (A226V) in the E1 protein has spread in areas where *Ae. albopictus* is predominant. This mutation increases the fitness of the virus for *Ae. albopictus* mosquitoes and its infectivity [8]. Disease outbreaks caused by the ECSA genotype have been reported in Europe. In particular, autochthonous outbreaks occurred in France in 2010, in 2014, and eventually in 2017 [9,10,11]. In 2007, an outbreak involving more than 200 cases was reported in Italy [12]. The virus showed the A226V mutation and index case was a viremic person from Kerala, India, visiting relatives in Italy. In 2017, a new outbreak of CHIKV was detected in Italy with three clusters of autochthonous transmission in the Lazio Region (central Italy), in the cities of Anzio, Rome, and Latina [13,14,15,16]. Afterwards, an additional cluster of transmission was detected in the city of Guardavalle Marina in the Calabria Region (south of Italy) [17]. As for the 21 December 2017, (http://www.salute.gov.it/portale/temi/documenti/chikungunya/bollettino_chikungunya_ULTIMO.pdf), the outbreak involved 192 confirmed cases from the Lazio Region and 74 from the Calabria Region. The median age was 54 years (range, 0–97 years). One patient died. The phylogenetic analysis showed that the virus involved in the Lazio outbreak belongs to the ECSA clade, and clusters within the Indian Ocean Lineage (IOL). *E1* sequences lacked the A226V substitution associated with increased viral fitness in *Ae. albopictus* [13,14].

Given the climate characteristics of Italy, the activity of the vector mosquitoes is mainly restricted to the summer season (usually between June and October); although the eggs could easily go overwintering, CHIKV has not shown transovarial transmission in *Aedes* spp. mosquitoes. Thus, sporadic outbreaks mostly driven by imported cases followed by autochthonous transmission are possible during the summer season.

Hence, outbreaks could be triggered by the arrival of imported cases from endemic areas and may result in secondary cases. No specific treatment or vaccine is yet available to prevent CHIKV infection and the containment of potential outbreaks mainly relies on the interruption of the transmission chain by well-designed preventive and reactive strategies to reduce the mosquitos’ density.

This highlights the importance of a well-designed surveillance system that should promptly identify autochthonous transmission. Several surveillance tools, using different data sources, and streams, have been developed, including passive surveillance, laboratory-based surveillance, syndromic surveillance, novel data streams, and entomological surveillance. Here we discuss the challenges of different surveillance tools and their possible application and integration based on the experience of the 2017 CHIKV outbreak in Lazio Region in Italy.

## 2. Passive Case Surveillance

Main objectives of the passive surveillance are to monitor imported cases, (in particular in areas where suitable mosquitoes are present) to assess the risk of possible autochthonous transmission and to identify early local transmission (diffusion, entity and term) in order to adapt public health measures (prevention and response activities) and to implement and supervise vector control activities. Passive case surveillance relies on hospitals, laboratories, and health practitioners notifying public health authorities of suspected or confirmed cases of infections occurring in the population. In Italy, since 2007, a national plan for the surveillance and control of CHIKV, dengue virus (DENV), and Zika virus (ZIKV) is released every year from the Ministry of Health before the summer season. Since 2015, Lazio Regional Health Authority has implemented an integrated surveillance system within the Italian national framework plan. In the Region, all suspected cases of arbovirus infection should be notified to the regional health authorities in order to promptly pre-activate the control measures. During the period 2015–2016, 244 suspected imported cases were reported to the Regional Service for Surveillance and Control of Infectious Diseases (SERESMI)—Lazio Region. Of these, 94 (38.5%) were confirmed or probable (only IgM positivity on a single sample) cases of arbovirus infection, including 45 (47.8%) DENV, 25 (26.6%) ZIKV, 20 (21.3%) CHIKV and 4 (4.3%) Toscana virus. The median time between symptom onset and notification was five days.

During 2017, only two imported CHIKV cases were notified before the detection of the outbreak in September, and vector control measures were promptly adopted. The two imported cases had no epidemiological link with the outbreak. The surveillance system was able to detect the first autochthonous case only after the peak of the outbreak [16] as it was in 2007 [12]. An active case finding was set up in the affected areas in order to retrospectively detect previous cases. All people reporting symptoms and signs during the previous five months were tested. Thus, early identification of autochthonous cases of CHIKV infection is the main challenge for a passive surveillance system. This is certainly due to the passive nature of the system, the under notification of cases, the knowledge of the physician, and the clinical features of CHIKV infection, where patients with mild symptoms often do not seek medical care. In a knowledge, attitude, and practice (KAP) study towards CHIKV among General Practitioners in the city of Rome carried out in 2012 [18], well before the 2017 outbreak, only 20% of the interviewees had knowledge about endemic areas of transmission, only 32% of the incubation period, and only 50% were aware of the risk of CHIKV introduction. In order to control the possible introduction and spread of the virus, in 2018 the national and Lazio regional plans have been amended. Following the national recommendation, the definition of a possible case (case with clinical symptoms and epidemiological link with an endemic country) has been established and vector control measures are initiated within 24 h after a possible case notification. Moreover, the Lazio Region has established a Regional Referral Clinical Center at the National Institute for Infectious Diseases “L.Spallanzani”, in Rome to guarantee the surveillance of clinical outcomes and the proper isolation measures of cases.

## 3. Laboratory-Based Surveillance

To face the increasing circulation of arboviruses, a network of regional reference laboratories for arboviruses has been established in Italy in the framework of the national surveillance plan.

The first active regional program for surveillance of arboviral infection started in summer 2007 to face an outbreak of the *Ae albopictus* -transmitted CHIKV in a narrow area of the Emilia-Romagna region [19].

In the Lazio Region, all the samples from suspected arboviral cases should be sent to the Regional Referral Laboratory at the National Institute for Infectious Diseases, “L.Spallanzani” (INMI) in Rome. In order to rapidly and efficiently identify any potential viral outbreak, and minimize as much the possible risks for public health, the priority is to have a continuous upgrade of diagnostic procedures, setup of diagnostic laboratory networks, and of course, a non-stop epidemiological surveillance.

The Regional Reference Laboratory guarantees the integrated surveillance of arboviruses transmitted by *Aedes* spp. Given the overlapping clinical presentation and epidemiological distribution of ZIKV, DENV, and CHIKV, this point is crucial. Overall, the diagnosis of arbovirus infections is challenged by the paucity or even lack of available commercial kits. Very often serological testing is the main diagnostic tool for arbovirus infections, due to short-lived viremia [20]. Test results interpretation may be difficult due to previous infections or vaccinations for similar viruses that may trigger the production of antibodies and lead to a misdiagnosis. Commercially available diagnostic tools may provide support and confirmation, but they cannot fully cover the response range of the highly complex diagnosis of arbovirus infections. Both USA and Europe have implemented similar strategies to detect arboviral infections and are continuously improving existing tests and searching for new ways to detect vector-borne pathogens. The maintenance of elevated standards of laboratory diagnosis needs trained personnel, and expert advice for correct interpretation. A rapid response and detection of emerging infections can be hampered by a shortage in validation panels and positive controls. Another critical area is the lack of a common platform for sharing validated panels. This analytical aspect is currently overcome thanks to the establishment of EU funded networks such as the ECDC Emerging viral disease expert laboratory network EVD-LabNet (https://www.evd-labnet.eu/) and the EU Joint Action EMERGE (http://www.emerge.rki.eu).

The diagnostic approach to arboviruses transmitted by *Aedes* spp. should be based on the differential diagnosis of acute febrile illnesses. In particular, in the presence of compatible clinical manifestations associated with a clear epidemiological link, it is crucial to perform specific molecular tests for each virus, accompanied by broader spectrum molecular tests for flaviviruses or alphaviruses. The same approach is followed in serological testing, for which, in the absence of positive tests based on the agent’s direct detection, it is absolutely necessary to test two samples at least at one week apart from each another. Moreover, neutralization tests against viruses’ members of same serotype and same geographic distribution should be considered in the presence of high cross-reactivity.

During the 2017, CHIKV outbreak regional health authorities identified the INMI Laboratory of Virology as the sole reference on the territory to better coordinate the control of the outbreak. All the results of diagnostic tests were reported to the SERESMI. This allowed the trigger of a rapid epidemiologic and entomologic investigation by authorities in order to activate all the control measures. The diagnosis was based on the detection of viral genome by real time RT-PCR (RealStar Chikungunya RT-PCR Kit 2.0, Altona Diagnostics GmbH, Hamburg, Germany) and virus-specific antibodies by serological tests (immunofluorescence assay, Anti-Chikungunya-Virus-IIFT (IgG/IgM), Euroimmun, Lübeck, Germany). Despite the long experience in the management of infectious emergencies, the outbreak strongly challenged the laboratory. From 7 September, when the first autochthonous CHIKV infection was confirmed, the workload increased by more than 80%. Only few cases (*n* = 7) with IgM positivity in the absence of both IgG and RT-PCR positive were critical for the CHIKV diagnosis. One of them showed a subsequent seroconversion. The other 6 cases were considered false positive. In these cases, paired sera were essential to well classify the cases.

## 4. Syndromic Surveillance

Syndromic surveillance was developed for early detection of a large-scale release of a biologic agent [21]. Syndromic surveillance is a systematic collection, analysis, and interpretation of information about patients’ symptoms during the early phases of illness. It can be used also to gather proxy data related to early illness such as school or work absenteeism, or veterinary data, etc. Here we focus on the use of syndromic surveillance to monitor the frequency of illnesses through a specific set of clinical features [22].

CHIKV infection is characterized by a low proportion (15%) of asymptomatic infections [23,24] and by the abrupt onset of high fever, severe arthralgia, myalgia and maculopapular rash. These signs and symptoms could be grouped in a dedicated syndrome. In the Lazio Region, 43 of the 47 total (91.5%) Emergency Departments (EDs) have an electronic registration platform (GIPSE) that can be consulted in real time, where signs, symptoms and diagnosis are registered for each access using the International Classification of Diseases—9th revision—Clinical Modification (ICD-IX-CM) codes, in addition to demographic data, management of access and outcome, history, and note.

To detect epidemic events, a syndromic surveillance system and statistical methods have been implemented by the Regional Service for Surveillance and Control of Infectious Diseases (SEREMI-Regione Piemonte) and SERESMI. The syndromes were defined as the presence of one or more codes from the ICD-IX-CM, related to the signs and the symptoms observed in EDs. Accordingly, this surveillance is less specific but much more sensitive than the regional passive reporting system (or regional specific surveillance system). Two statistical methods were adopted to detect the epidemic signal, computed daily-based and weekly-based, respectively. The daily based method compared the number (and proportion) of access to ED of the specific syndrome, with the expected number (or proportion) computed on the basis of seven days before. The Poisson confidence interval of each daily number (or proportion) of access to ED of the specific syndrome was compared with the expected one, the alert signal was detected if the observed value was bigger than expected and the confidence interval did not include the expected value. The epidemic signal was defined by more than two consecutive alert signals. The weekly based method consisted of the weekly amount of access in ED of the specific syndrome being analyzed by a periodic regression model. The alert signal was defined if the weekly observed number (or proportion) was bigger than the threshold. The epidemic signal was defined by more than two consecutive alert signals. The system is characterized by low specificity and high sensitivity compared with passive surveillance and can be used to integrate conventional surveillance systems.

During 2017, the system showed several alerts regarding the “Fever with rash” syndrome also during the summer season (when the 2017 CHIKV outbreak occurred). The alerts were referred to the concomitant ongoing outbreak of measles [25] and no additional investigation was conducted.

At the start of the 2018 high vector activity season, the SERESMI decided to include three new syndromes in the syndromic surveillance systems with focus on arboviruses, namely “CHIKV syndrome”, which include code related to arthralgia and myalgia; “CHIKV syndrome with fever”, which includes the previous codes and fever; “arboviruses syndrome”, which includes the codes related to arboviruses infections; and the “insect bite syndrome”. Codes are reported in the Supplementary Material. The new syndromes were retrospectively analyzed from January 2013 up to the 16 July 2018 and the data are provided below.

**Figure 1:**
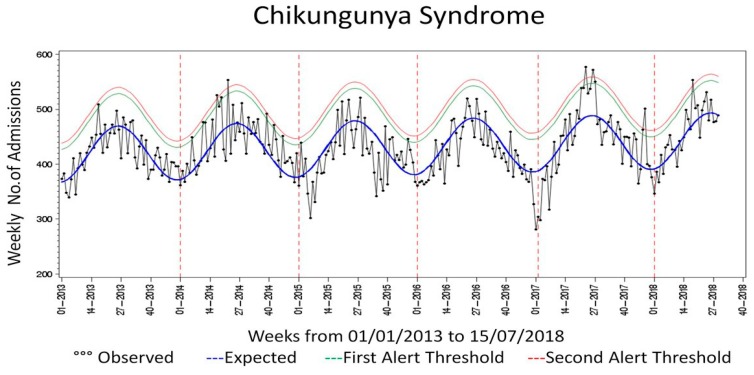
Number of Emergency Department (ED) admissions expected value and alert threshold for Chikungunya Virus (CHIKV) Syndrome. Blue Line, Expected number of ED admissions; Green Line, upper 95% confidence interval for the expected number of ED admissions (first alert threshold); Red Line, upper 99% confidence interval for the expected number of ED admissions (second alert threshold).

**Figure 2:**
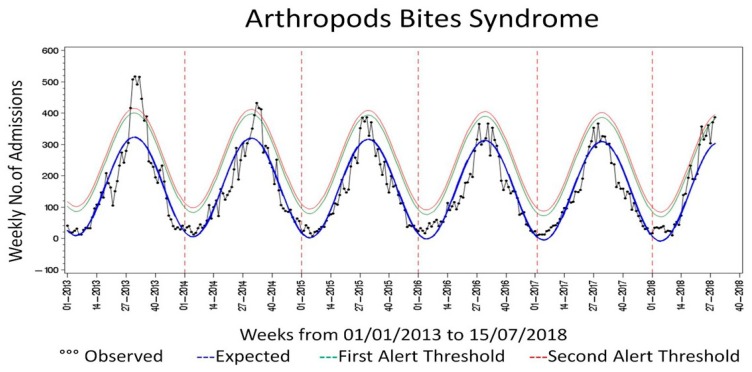
Number of ED admissions, expected value and alert threshold for Arthropod bites syndrome. Blue Line, Expected number of ED admissions; Green Line, upper 95% confidence interval for the expected number of ED admissions (first alert threshold); Red Line, upper 99% confidence interval for the expected number of ED admissions (second alert threshold).

Probably due to the high underreporting of fever during the ED admission, the “CHIKV syndrome with fever” has not produced reliable results. As shown in Figure 1, applying the new “CHIKV syndrome” to the previous year, two alerts are present during the 2017 summary. No alert was present for the “CHIKV syndrome with fever”. Regarding the arthropod bites syndrome (Figure 2), the seasonality in the frequency of insect bites is clearly shown, which peaks in the summer period.

## 5. Non-Traditional Data Sources

The use of novel data streams (NDS), such as search generated data or social media updates are a promising approach in enhancing and/or complementing a traditional surveillance system [26,27] and informing/supporting public health actions [28]. Systematically monitoring, collecting, and analyzing social media data and web searches could have the potential not only to predict events relevant for public health purposes but also to investigate the effect of media coverage as potential misinformation and biases (so called “epidemic of fear”) [29]. Following the H1N1 pandemic of 2009, 2 million messages on Twitter (“tweets”) related to the pandemic were analyzed [30]. It was concluded that these messages could be used for real-time content analysis, which could potentially allow health authorities to deal with public concerns. The same conclusion was drawn during the Ebola outbreak [31]. In a systematic review on the use of novel data streams for tropical and subtropical communicable diseases [32], 24 studies were related to arbovirus infections with the majority (16) of them exploring the use of NDS for ZIKV and one for CHIKV infection. In the latter study [33], the Twitter monitoring was used to integrate a spatio-temporal model as a proxy of human behavior against mosquitoes during the 2014 outbreak in Martinique. The reaction of the 2017 outbreak in Italy has been analyzed in terms of Google Trends, Google News, Twitter traffic, Wikipedia visits and edits, and PubMed articles, exploiting structural modelling equations [34]. A significant correlation was observed between the search volume on Google trends, the number of tweets on Twitter, and notified cases (*p* = 0.0008 and 0.0171, respectively); no significant correlation was observed with Wikipedia edits (*p* = 0.0524). The significant public reaction documented could be used to guide public health authorities to address the population’s concerns though disseminating awareness and avoid misleading information. The use of this novel data to detect outbreaks earlier should be further studied. At the moment the use of these surveillance systems is under evaluation in our context.

## 6. Entomological Surveillance

The two main CHIKV vectors are *Ae aegypti* and *Ae albopictus*. Both have adapted to the urban environment, where they colonize artificial water containers (e.g., Buckets, broken crockery, flower vases, tires, or water storage drums). *Ae aegypti* is primarily endophilic (indoor) and bites at daytime and crepuscular time [35]. *Ae. albopictus* generally lives in peridomestic areas and is mainly a diurnal outdoor biter, and mainly breeds in outdoor storage and discarded containers. *Ae albopictus* is a highly invasive mosquito and has greatly expanded into temperate areas [36]. *Ae albopictus* colonies were reported in Genoa, northern Italy, in 1990 [37] and in the Veneto Region in the following year. The source of the infestation was confirmed to be the trade of used tires from the USA [38]. The species has rapidly spread throughout the country with scattered foci mainly in inhabited [39] areas. *Ae. albopictus* has been responsible for the outbreak of CHIKV that occurred in Italy in 2007, which was the first in Europe [12] and for the 2017 outbreak in the Lazio region [40]. Vector surveillance and control share the objective to monitor and reduce vector density in order to not allow epidemic transmission. Mosquitoes are collected, identified, pooled by species, and tested for virus infections. There are different strategies for mosquito collection. Current approaches collect immature stages (larvae or pupae) and adults. The collection of larvae and pupae is used as a proxy of vector density and data is used to calculate several indices of risk [41]. Most of these indices have not yet been validated for assessing the risk of CHIKV amplification [42]. Adult collection has different sampling strategies and uses different traps. It is used to estimate the population involved in the transmission and to detect viral activity. Up to 2017, in the Lazio Region there has been no systematic entomological surveillance of *Aedes* spp. involving mosquito collection and risk assessment. Following the 2017 outbreak and the recommendation of the National Surveillance Plan, in 2018 efforts are directed to performing a targeted surveillance of the so called “hotspots”, which are identified on the basis of the environmental and epidemiological characteristics of the areas. The first hot spot monitoring network of the *Ae albopictus* mosquito has been established in the provincial capitals and in the metropolitan area of Rome. The surveillance involves the collection of immature stages and adults.

## 7. Conclusions

Systematic reviews highlight the critical characteristics of an efficient alert system, which should be sensitive enough to predict or detect outbreaks, specific enough to avoid false alerts, and timely enough to adopt control measures [43].

Passive surveillance systems may suffer from the underreporting of cases. An analysis of data from the US National Notifiable Disease Surveillance System (NNDSS) from 2004 through 2016 showed a substantial underestimation of neuroinvasive and nonneuroinvasive arthropodborne viral (arboviral) disease occurrences [44]. This highlights the need to promote the specific capacity-building of health practitioners toward vector borne diseases as a necessary step to establish an efficacious surveillance system in a non-endemic country. An educational plan should target ED physicians, General Practitioners (GPs), pediatricians, and infectious diseases specialists, covering the management of the whole spectrum of arboviruses. Educational programs should prioritize the epidemiological aspects of the diseases and related risk of introduction and spread, the clinical and diagnostic profile, and prevention and control measures. Given the changes in the epidemiological profiles, a higher coverage of these diseases during the academic education of health science schools should be also planned. The laboratory surveillance should be a key component of epidemiological surveillance, also providing a tool for reducing the underreporting of cases. Passive surveillance is often enhanced in endemic countries through the establishment of multiple approaches, such as household-based cluster investigation, facility based surveillance, and the enhanced detection of fatal cases [45]. Given the epidemiological profile of Italy, with seasonal vector activity, such an enhanced surveillance could be established once an autochthonous outbreak occurs. The syndromic surveillance can be used as an additional tool to monitor the trend and to evaluate alerts regarding CHIKV infection. The interpretation of data has several limitations, which are the natural limitations of a syndromic surveillance system. The system lacks specificity and the alerts of “chikungunya” syndrome could be referred to other non-communicable diseases, such as rheumatological diseases that show a recrudescence during the hot seasons. Moreover, the monitoring of the EDs admission would decrease the sensitivity of the system in light of the CHIKV clinical signs and symptoms, which often do not require a referral to the ED. The expansion of the system to GPs could increase the sensitivity. Regarding the arthropod bites syndrome, the system could be used to draw a baseline incidence and this could be used to monitor the seasonality of the vector activity and the impact of weather events and climate change [46]. Novel data sources, could be an important tool to integrate traditional surveillance and to support public health authorities in collecting public concerns and replying to them, disseminating awareness, and avoid misleading information. This could also inform the entomological surveillance through proxy data regarding human behavior and vector activity. The information exchange at the institutional level is key in assessing the risk and evaluating possible prevention measures.

Empirical evidence from other countries with a temperate climate have highlighted the importance of an integrated surveillance system. Since 2006, France has established an integrated preparedness and response plan that includes epidemiological and entomological surveillance [47]. The plan has been challenged by the increasing number of imported cases, which strengthens the need for addition sources of information and better knowledge and education of the population and health professionals.

The possible spread of other viruses transmitted by *Aedes* spp., such as DENV and ZIKV, should be also addressed by the different surveillance tools described. Additional routes of transmission and clinical complications should be taken into account integrating the system with systematic clinical follow up cases (DENV, ZIKV) and with congenital malformation registers (ZIKV).

In conclusion, the integration of different surveillance tools and the combination with entomological surveillance in a one health dedicated surveillance system should facilitate the detection, response, and control of arbovirus spreading [48], including CHIKV. The integration between human surveillance and entomological surveillance, in a one-health perspective is key in adapting control measures, providing continuous risk assessment and promptly recognizing an outbreak. Both surveillance components should be strengthened using new surveillance tools such as syndromic surveillance and novel data sources.

## Supplementary Materials

The following are available online at http://www.mdpi.com/1999-4915/10/9/501/s1.
