# Supplementary material for: The Surveillance of Chikungunya Virus in a Temperate Climate: Challenges and Possible Solutions from the Experience of Lazio Region, Italy
Source: Viruses. 2018 Sep 14;10(9):501. doi: 10.3390/v10090501 (PMC6163295; doi:10.3390/v10090501)
Supplement: Supplementary file 1 [file viruses-10-00501-s001.pdf]

CHIKUNGUNYA SYNDROME	
**At least one of the following**	
782.1	Rash and other non-specific skin rashes
719.4	Joint pain
719.40	Arthralgia, unspecified site
719.41	Arthralgia, shoulder
719.42	Arthralgia, elbow
719.43	Arthralgia, wirst
719.44	Arthralgia, carpus, metacarpus and interfalangeal joints of the hand
719.45	Arthralgia, hip
719.46	Arthralgia, knee
719.47	Arthralgia, tibio-tarsic and joints of the foot
719.48	Arthralgia, other specified locations
719.49	Arthralgia, multiple sites
711	Arthropathy from infectious agents
711.9	Infectious arthritis unspecified<br>Arthritis or infectious polyarthritis (acute) (chronic) (subacute) SAI
711.90	Infectious arthritis, unspecified, location not specified
711.91	Infectious, unspecified arthritis, shoulder
711.92	Infectious arthritis unspecified, Elbow
711.93	Infectious arthritis, unspecified, wirst
711.94	Unspecified infectious arthritis, carpus, metacarpus and interphalangeal joints of the hand
711.95	Infectious arthritis, unspecified, hip
711.96	Infectious infectious arthritis, knee
711.97	Infectious, unspecified arthritis, tibio-tarsic and joints of the foot
711.98	Infectious arthritis unspecified, others specified locations
711.99	Infectious infectious arthritis, multiple locations
**ARBOVIROSES SYNDROME**<br>**At least one of the following**	
061	Dengue
065	Hemorrhagic fever by arthropods
065.0	Crimea-Congo haemorrhagic fever
065.1	Hemoglobic fever of Omsk
065.2	Kyasanur forest disease
065.3	Other tick-borne fever
065.4	Hemorrhagic fever by mosquitoes
065.8	Other hemorrhagic fevers by arthropods, specified
065.9	Hemorrhagic fevers by arthropods, unspecified
066.3	Other mosquito fevers
066.4	West Nile Fever
066.40	West Nile Fever unspecified
066.41	West Nile Fever with Encephalitis
066.42	West Nile Fever with other neurological

	complications
066.49	West Nile Fever with other complications
062	Viral mosquito encephalitis
062.0	Japanese encephalitis
062.1	West type equine encephalitis
062.2	Equine encephalitis type est
062.3	Encephalitis of St. Louis
062.4	Australian encephalitis
062.5	California virus encephalitis
062.8	Other viral mosquito encephalitis, specified
060	Yellow fever
062.9	Viral mosquito encephalitis, unspecified
063	Tick-borne viral encephalitis
063.0	Russian spring-summer encephalitis [taiga]
063.1	Louping ill
063.2	Central European encephalitis
063.8	Other viral tick-borne encephalitis, specified
063.9	Tick-borne viral encephalitis, unspecified
064	Viral encephalitis transmitted by other and unspecified arthropods
066	Other viral diseases from arthropods
066.0	Phlebotomist fever
066.1	Tick fever
066.2	Equine fever of Venezuela
**ARTHROPOD BITE SYNDROME<br>At least one of the following**	
E906.4	Bite of non-poisonous arthropod
910.4	Arthropod bite, not poisonous, without mention<br>of face, neck and scalp infection, excluding the eye
910.5	Arthropod bite, non-poisonous, with face, neck and scalp infection, excluding the eye
911.4	Arthropod bite, not poisonous, without mention<br>Of trunk infection
911.5	Arthropod bite, not poisonous, with trunk infection
912.4	Arthropod bite, not poisonous, without mention<br>of shoulder and arm infection
912.5	Arthropod bite, non-poisonous, with shoulder and arm infection
913.4	Arthropod bite, not poisonous, without mention

	of elbow, forearm and wrist infection
913.5	Arthropod bite, non-poisonous, with elbow, forearm and wrist infection
914.4	Arthropod bite, not poisonous, without mention of hand infection
914.5	Arthropod bite, not poisonous, with hand infection
915.4	Arthropod bite, not poisonous, without mention of infection fingers of the hand
915.5	Arthropod bite, non-poisonous, with hand fingers infection
916.4	Arthropod bite, not poisonous, without mention of infection of the hip, thigh, leg and ankle
916.5	Arthropod bite, non-poisonous, with infection of the hip, thigh, leg and ankle
917.4	Arthropod bite, not poisonous, without mention of infection of the foot and toes
917.5	Arthropod bite, non-poisonous, with infection of the foot and toes
919.4	Arthropod bite, not poisonous, without mention of infection of other, multiple and unspecified locations
919.5	Arthropod bite, non-venomous, with infection of other, multiple and unspecified locations
